# Case Report: Acute prostatic swelling following hydrogel spacer insertion managed by MR-guided online adaptive radiotherapy

**DOI:** 10.3389/fonc.2026.1905108

**Published:** 2026-09-16

**Authors:** Yushi Takahashi, Noriyoshi Takahashi, Sakiko Kageyama, Shohei Tanaka, Rei Umezawa, Takaya Yamamoto, Yu Suzuki, Keita Kishida, So Omata, Keiichi Jingu

**Affiliations:** 1 Department of Radiation Oncology, Tohoku University Graduate School of Medicine, Sendai, Japan; 2 Department of Diagnostic Radiology, Tohoku University Hospital, Sendai, Japan

**Keywords:** case report, hydrogel spacer, MR-guided adaptive radiotherapy, prostate cancer, prostate swelling, stereotactic body radiotherapy

## Abstract

**Background:**

Hydrogel spacers are widely used in radiotherapy for localized prostate cancer to reduce the rectal dose. While generally safe, acute anatomical changes in the prostate gland itself, specifically significant swelling, are poorly described and can compromise the precision of radiotherapy.

**Case presentation:**

We report a case of a 62-year-old man with high-risk prostate cancer (PSA 15.9 ng/mL, Gleason score 4 + 4, cT2cN0M0) who underwent hydrogel spacer placement for stereotactic body radiotherapy (SBRT) (40 Gy in 5 fractions). Although magnetic resonance imaging (MRI) for radiation planning on day 8 postinsertion revealed a normal prostate volume (22.0 cc), pretreatment MRI for MR-guided online adaptive radiotherapy (MRgOART) on day 23 (at the second fraction) revealed a peak volume of 31.4 cc, a 42.7% increase. This swelling compromised the dosimetry of the initial plan, risking insufficient coverage of the clinical target volume (CTV). Using MRgOART, the plan was successfully reoptimized to prevent target underdosing.

**Conclusions:**

We report a rare instance of acute prostate swelling following hydrogel spacer insertion. Although the precise etiology remains unconfirmed, such an anatomical change, if unrecognized, could degrade treatment accuracy. This case demonstrates that online adaptive radiotherapy via an MR-Linac is essential for detecting and managing unexpected anatomical variations during prostate SBRT.

## 1 Introduction

Stereotactic body radiotherapy (SBRT) is a standard treatment option for localized prostate cancer, offering biochemical control rates comparable to those of surgery (1–4). To avoid radiation-induced rectal injury, polyethylene glycol (PEG)-based hydrogel spacers are widely used to separate the prostate from the rectum (5). Pivotal trials and meta-analyses have confirmed that spacers reduce rectal irradiation and late toxicity (6–8).

While generally safe, spacer insertion carries risks. The complications documented in the Manufacturer and User Facility Device Experience (MAUDE) database range from rectal wall infiltration to rare abscesses or fistulas (9). Although prostate volume is typically assumed to be stable between insertion and treatment (10, 11), we present a rare case of acute and significant prostate swelling following hydrogel spacer insertion that was successfully managed with magnetic resonance-guided online adaptive radiotherapy (MRgOART).

## 2 Case description

A 62-year-old man with hypertension, dyslipidemia, and hyperuricemia was referred for National Comprehensive Cancer Network (NCCN) high-risk localized prostate cancer (prostate-specific antigen (PSA) 15.9 ng/mL; Gleason score 4 + 4 = 8; cT2cN0M0 on computed tomography (CT), magnetic resonance imaging (MRI) and bone scintigraphy) (12). Diagnostic multiparametric MRI revealed a 26-mm lesion in the peripheral zone (**Supplementary Figure 1**).

Treatment consisted of six months of androgen deprivation therapy (ADT) with oral bicalutamide (80 mg once daily) and leuprorelin acetate followed by SBRT (40 Gy in 5 fractions) with a hydrogel spacer. The spacer (SpaceOAR™, Boston Scientific, Marlborough, MA) was inserted transperineally under ultrasound guidance via local anesthesia. Postprocedure ultrasound confirmed correct placement with no immediate swelling.

MRI for planning on day 8 postinsertion revealed a prostate volume of 22.0 cc with no signal abnormalities (**Figure 1A**). An SBRT plan was generated via the Monaco™ system for 1.5 Tesla magnetic resonance linear accelerator (MR-Linac, Elekta AB, Stockholm, Sweden). Treatment was delivered using 7 MV flattening filter-free (FFF) photon beams with a step-and-shoot intensity-modulated radiotherapy (IMRT) technique (9 coplanar fields). The clinical target volume (CTV) included the prostate and proximal 1.5 cm of the seminal vesicles. The planning target volume (PTV) was defined by adding a 5-mm margin to the CTV. The prescribed dose was 40 Gy to the CTV (excluding the urethra) and 36.25 Gy to the PTV (excluding overlap with the rectum, urethra, and bowel), delivered in 5 fractions every other day, excluding weekends and public holidays. The treatment plan met almost all of our institutional dosimetric constraints. The full institutional dosimetric protocol is provided in **Supplementary Table 1**. Prostate volumes at each time point were measured through manual contouring by a radiation oncologist on T2-weighted MRIs.

**Figure 1:**
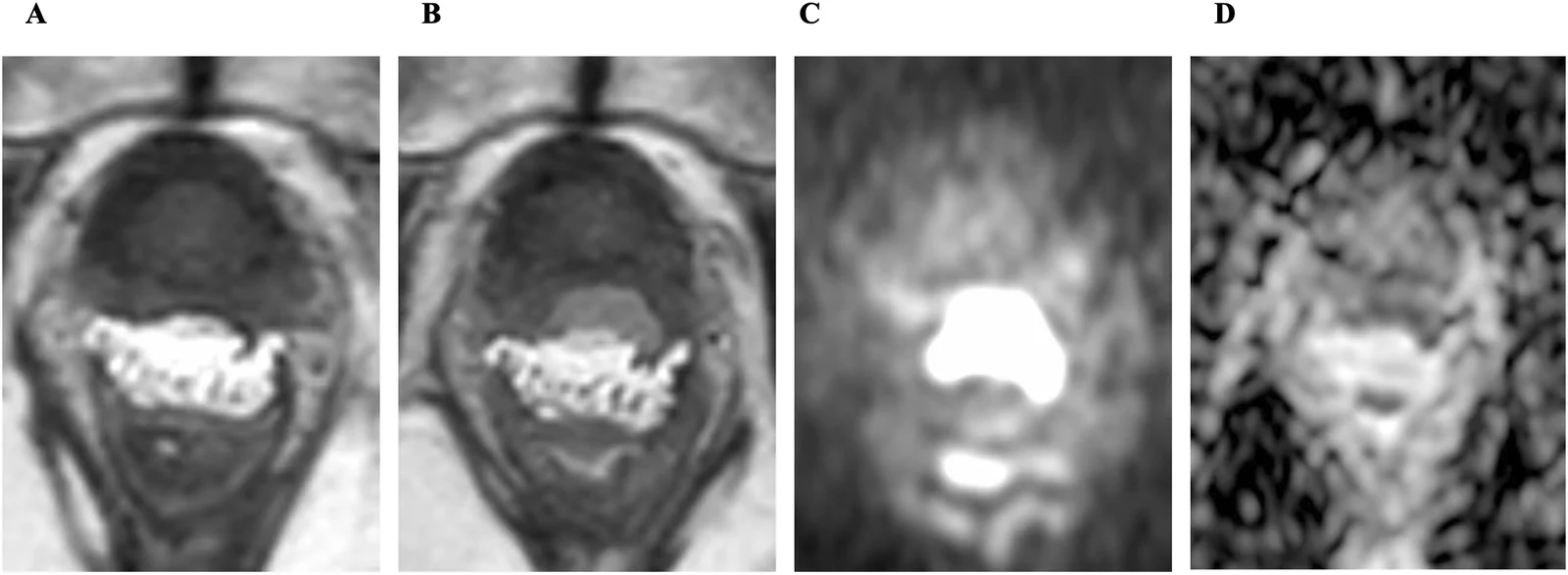
Comparison of prostate anatomy between planning and treatment. **(A)** The planning T2-weighted image on day 8 postinsertion shows a normal prostate gland. **(B)** T2-weighted image on day 23 postinsertion reveals marked swelling with a high-signal intensity area indicating inflammation and mild rectal wall thickening. **(C)** DWI (b = 1000 s/mm²) and **(D)** ADC map on day 23 postinsertion confirm restricted diffusion in the corresponding high-signal area.

On day 21 postinsertion, prior to delivering fraction 1, MRI for online adaptive radiotherapy revealed an unexpected volume increase to 25.1 cc. T2-weighted imaging demonstrated a new hyperintense area adjacent to the spacer (**Supplementary Figure 2**). These changes preceded any radiation. By day 23 postinsertion (fraction 2), both volumetric and signal abnormalities had further progressed. Prostate volume peaked at 31.4 cc (42.7% increase relative to the planning baseline), accompanied by expanding T2-weighted hyperintensity (**Figure 1B**). Imaging findings progressed, demonstrating restricted diffusion on diffusion-weighted imaging (DWI) and apparent diffusion coefficient (ADC) maps (**Figures 1C, D**) consistent with acute inflammation for the first time. Mild rectal wall thickening was also noted.

This acute swelling significantly decreased target coverage when the initial plan was applied to the anatomy of fraction 2 (day 23 postinsertion) (**Figure 2A**). Without adaptation, target coverage would have compromised significantly: CTV D95% dropped from the prescribed > 40 Gy to 37.19 Gy (a 7.0% reduction), PTV D95% from ≥ 36.25 Gy to 30.65 Gy (a 15.4% reduction), and PTV D98% from > 34.45 Gy to 27.50 Gy. However, online re-optimization via the MRgOART workflow restored CTV D95% to 39.42 Gy and PTV D95% to the prescribed 36.25 Gy, while maintaining all organs at risk constraints (**Figure 2B**). A detailed dosimetric comparison is shown in **Table 1**.

**Figure 2:**
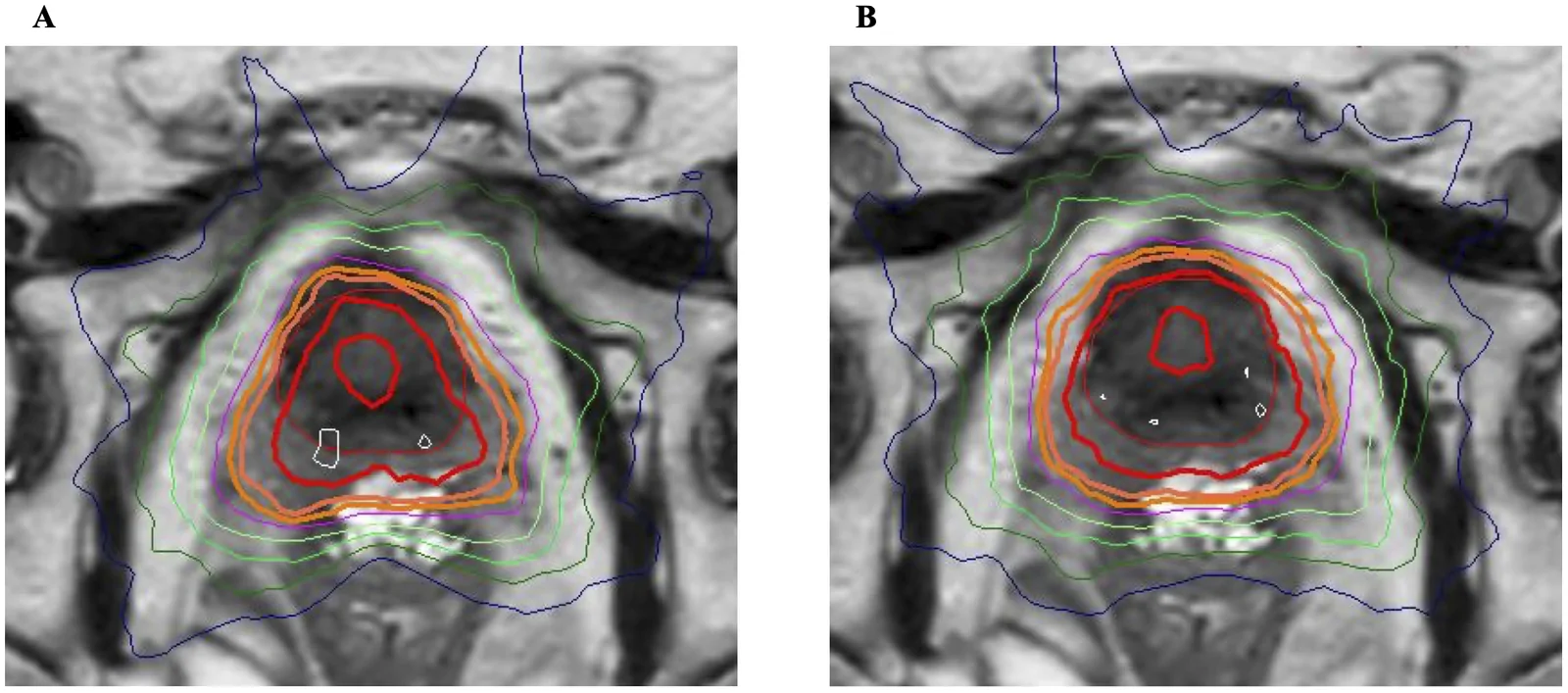
Dose distribution on the second day of treatment. **(A)** When the initial treatment plan is applied to the anatomy of fraction 2 (day 23 postinsertion), the anterior part of the CTV (narrow red line) is outside the prescribed dose. **(B)** With online adaptive replanning, the CTV coverage is appropriately restored.

**Table 1:** Dosimetric comparison of the initial plan versus the adapted plan for the anatomy of fraction 2 (day 23 postinsertion).

Structure	Dose metric	Constraint	Tolerance	Initial plan (day 23)	Adapted plan (day 23)	
**CTV†**	D95%	> 40.00 Gy	> 39.20 Gy	37.19 Gy *	39.42 Gy	
**PTV††**	D95%	≥ 36.25 Gy	N/A	30.65 Gy *	36.25 Gy	
	D98%	> 34.45 Gy	> 32.63 Gy	27.50 Gy *	35.28 Gy	
**Rectum**	Dmax	< 40.00 Gy	N/A	30.26 Gy	26.84 Gy	
	V36 Gy	< 1.0 cc	N/A	0.0 cc	0.0 cc

Dxx%, dose covering xx% of the target volume; Vxx Gy, volume receiving at least xx Gy; Dmax, maximum point dose. †CTV excluding the urethra. ††PTV excluding overlap with the rectum, urethra, and bowel. *Bold values indicate target coverage failing to meet the dosimetric constraints including tolerance.

The patient completed all five SBRT fractions without interruption using daily adaptation. The prostate volume began to decrease from the third day of treatment and returned to 25.1 cc by the end of the treatment course. The temporal changes in prostate volume and the key clinical events throughout the treatment course are summarized in **Figure 3**, and the sequential T2-weighted MRI obtained from each fraction, demonstrating the gradual resolution of the prostatic edema, is shown in **Supplementary Figure 2**. At 1-year follow-up, the PSA level declined to <0.006 ng/mL, with no evidence of biochemical or local recurrence.

**Figure 3:**
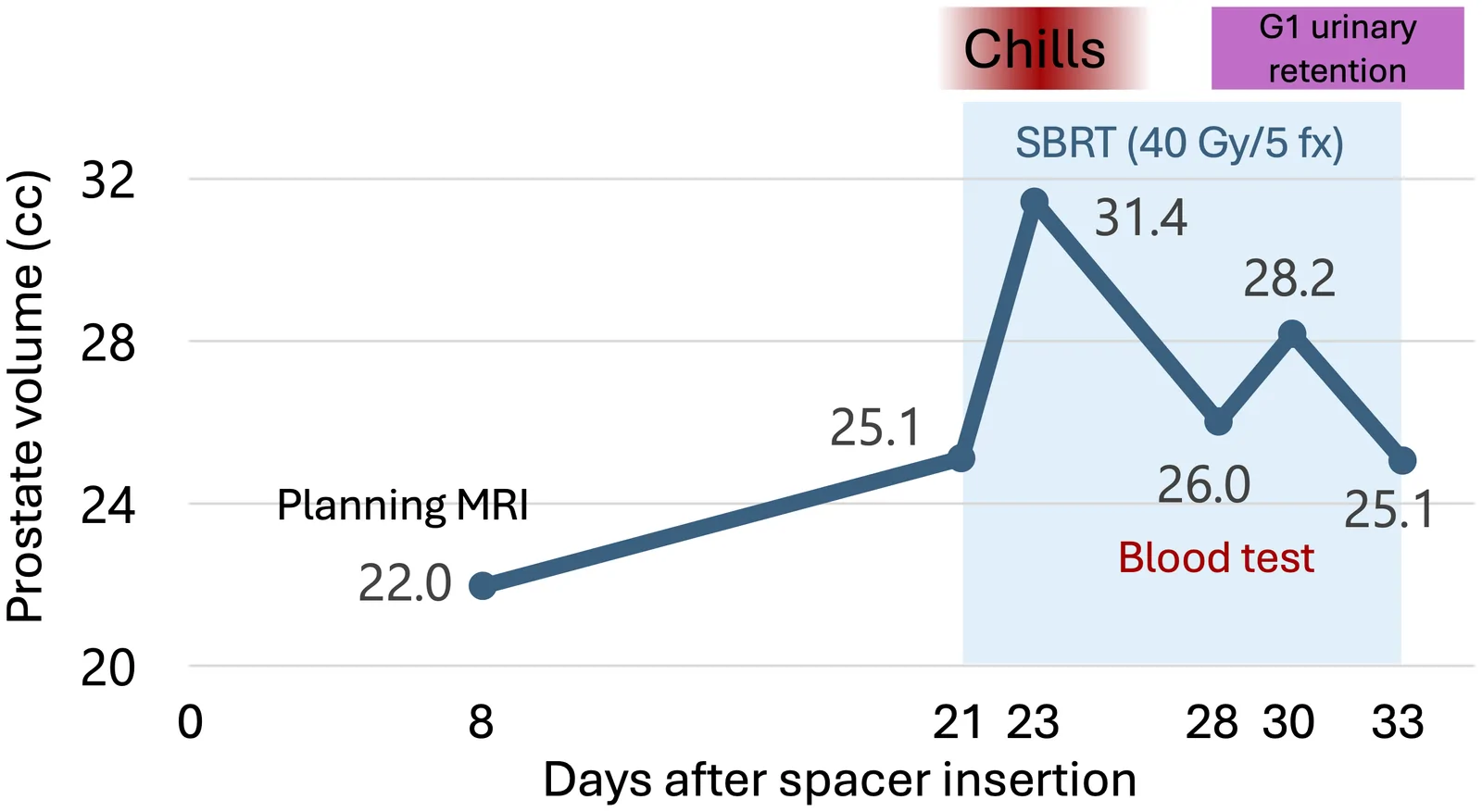
Timeline of prostate volume changes and key clinical events over the treatment course. Volume peaked at fraction 2 (day 23; 31.4 cc, representing a 42.7% increase from the 22.0 cc planning baseline) before returning toward baseline by the completion of treatment. Key clinical milestones are annotated along the timeline.

Adverse events were graded according to the Common Terminology Criteria for Adverse Events (CTCAE) version 5.0. During treatment, the patient reported chills during the first two fractions but remained afebrile. Grade 1 urinary retention developed from the third fraction. Blood tests on the day of the third fraction (day 28 postinsertion) revealed mild leukocytosis (white blood cell count (WBC) 9,600/µL). However, C-reactive protein (CRP) was only marginally elevated to 0.44 mg/dL (reference < 0.14 mg/dL). Serum immunoglobulin E (IgE) levels were within normal limits, and PSA levels remained stable compared with those at baseline (**Supplementary Table 2**). Given the absence of fever, minimal inflammatory marker elevation, and spontaneous resolution of the swelling, blood and urine cultures were omitted, and antibiotics were withheld. Clinical assessment relied on routine blood work and daily MR images acquired during the online adaptive workflow. Two weeks after the completion of SBRT, Grade 2 urinary frequency and Grade 1 hematochezia were observed. Regarding late toxicity, Grade 1 urinary incontinence developed approximately 10 months post treatment and was still present at the 1-year follow-up. No Grade 2 or higher late gastrointestinal toxicity was observed. These events were subsequently managed carefully, and no events of Grade 3 or higher occurred.

## 3 Discussion

This report describes a rare case of marked acute prostate swelling (>40%) developing after hydrogel spacer insertion tracked serially throughout treatment. While hydrogel spacer related complications like infection and urinary symptoms have been reported previously, similar volumetric events may be embedded within those reports. To our knowledge, this is the first report to document the complete onset-to-resolution trajectory of acute peri-treatment prostatic swelling using daily volumetric and multiparametric MRI. While radiation-induced prostate swelling during SBRT typically progresses to a peak at fraction 3, 4 or 5 with a mean volume increase of 11–21% (13–15), our case is distinguished by its greater magnitude (a 42.7% increase) and exceptionally early peak at fraction 2.

The etiology of this swelling is likely multifactorial and cannot be definitively established. Crucially, both the volume increase (25.1 cc vs. 22.0 cc at planning) and MRI inflammatory changes were already evident prior to fraction 1 on day 21. Given that the planning MRI on day 8 postinsertion revealed no signal abnormalities, this process clearly developed between one and three weeks after spacer insertion, indicating the definitive presence of a radiation-independent component. This timeline rules out radiation-induced edema as the sole cause. Several non-radiation etiologies were considered. Mild chills suggested a systemic response, yet the absence of fever and only marginal CRP elevation made a frank bacterial abscess unlikely. Rapid resolution argued against tumor progression. Normal IgE levels did not support an immediate hypersensitivity reaction. The concurrent use of ADT may have obscured PSA-based assessment.

A sterile inflammatory response to the hydrogel spacer is one plausible contributor. Although spacer-related complications (e.g., prostatitis, abscess) are documented (9), they were clinically overt and required intervention. Conversely, the prostatic swelling in our case was subclinical, self-limiting, and identified solely through daily MRI. Such pronounced, asymptomatic parenchymal enlargement three weeks post-insertion appears to be rarely documented, as reported spacer toxicities typically involve focal periprostatic or rectal pathology rather than diffuse prostatic edema. Such swelling may be underrecognized and embedded within registry data (e.g., MAUDE) lacking serial imaging. Daily MRI thus likely unmasked an underappreciated entity rather than a unique anomaly. We hypothesize that a pre-irradiation spacer reaction was compounded by early SBRT-induced edema, causing a cumulative, greater-than-expected volume increase. Accompanying rectal wall thickening at fraction 2 aligns with this local inflammation, though the relative contributions of the spacer and radiation remain unconfirmed.

Regardless of the precise cause, this swelling compromised the initial dosimetry. Given the low α/β ratio and the dose sensitivity of prostate cancer (16), such an unintended CTV underdose—especially in high-risk disease—could compromise local control. MRgOART was essential for detecting this silent change and restoring target coverage via online adaptation. This case underscores the value of online adaptive radiotherapy in managing unforeseen anatomical changes. For centers relying on cone-beam computed tomography (CBCT), which has limited soft-tissue contrast, relying solely on fiducial markers risks missing such volumetric changes. Careful verification of the prostate shadow or confirmatory imaging is recommended when anatomical discrepancy is suspected. Therefore, MRgOART via an MR-Linac may offer particular advantages for detecting and compensating for unexpected volumetric changes during treatment.

This report is limited by its single-case nature, and the precise etiology of the swelling could not be pathologically confirmed. Further studies are needed to determine the incidence and risk factors for delayed prostatic swelling following hydrogel spacer insertion.

## 4 Conclusions

We experienced a rare case of acute prostate swelling, likely induced by hydrogel spacer insertion. Such an anatomical change, if unrecognized, could degrade treatment accuracy. This case highlights the critical role of online adaptive radiotherapy via an MR-Linac in ensuring accurate dose delivery when unexpected anatomical changes occur during treatment.

## Data Availability

The original contributions presented in the study are included in the article/**Supplementary Material**. Further inquiries can be directed to the corresponding author.
