# Supplementary material for: Case Report: Acute prostatic swelling following hydrogel spacer insertion managed by MR-guided online adaptive radiotherapy
Source: Front Oncol. 2026 Sep 16;16:1905108. doi: 10.3389/fonc.2026.1905108 (PMC13623546; doi:10.3389/fonc.2026.1905108)
Supplement: Supplementary file 1 [file DataSheet1.docx]

## **Supplementary Material**

### A B C

**Supplementary Figure 1.** **Diagnostic MRI of the prostate.** **A** T2-weighted image shows a low-signal intensity lesion extending from the left to the right peripheral zone without extracapsular extension. **B** DWI (b = 1500 s/mm²), and **C** ADC map demonstrate restricted diffusion in the same region.

### A B C D E

**Supplementary Figure 2. Sequential changes in prostate anatomy during the treatment course.** Daily pretreatment T2-weighted MRI demonstrating the gradual resolution of acute prostatic edema over time. **A** Fraction 1. **B** Fraction 2. **C** Fraction 3. **D** Fraction 4. **E** Fraction 5.

**Supplementary Table 1.** Institutional dosimetric protocol and constraints for prostate SBRT

| Structure | Dose Metric | Constraint | Tolerance |
| --- | --- | --- | --- |
| CTV† | D95% | > 40.00 Gy | > 39.20 Gy |
| CTV | D98% | > 36.25 Gy | N/A |
| PTV†† | Dmax | < 43.20 Gy | N/A |
|  | D95% | ≥ 36.25 Gy | N/A |
|  | D98% | > 34.45 Gy | > 32.63 Gy |
| Rectum | Dmax | < 40.00 Gy | N/A |
|  | V36 Gy | < 1.0 cc | N/A |
|  | V32.6 Gy | < 15% | N/A |
|  | V29 Gy | < 20% | N/A |
|  | V25.3 Gy | < 30% | N/A |
|  | V21.7 Gy | < 40% | N/A |
|  | V18.1 Gy | < 50% | N/A |
| Bladder††† | V37 Gy | < 5.0 cc | < 10 cc |
|  | V18.1 Gy | < 40% | < 50% |
| Bowel | D0.5 cc | < 33.00 Gy | N/A |
|  | D1.0 cc | < 30.00 Gy | N/A |
|  | V25 Gy | < 10 cc | < 20 cc |
|  | V20 Gy | < 20 cc | < 30 cc |
| Femur (L/R) | Dmax | < 25.30 Gy | N/A |
|  | V14.5 Gy | < 5% | N/A |
| Urethra | Dmax | < 40.00 Gy | N/A |

*Abbreviations: Vxx Gy, volume receiving at least xx Gy; Dmax, maximum point dose; Dxx%, dose covering xx% of the volume; Dxx cc, dose covering xx cc of the volume. †CTV excluding the urethra. ††PTV excluding overlap with the rectum, urethra, and bowel. †††Bladder excluding overlap with the prostate.*

**Supplementary Table 2.** Blood test data during the treatment period.

| Category | Test Item | Unit | Day 0 | Day 28 | Reference Range |
| --- | --- | --- | --- | --- | --- |
| Hematology | **WBC** | /µL | 4,100 | 9,600 | 3,300–8,600 |
|  | **Neutrophils** | % | 46.5 | 56.3 | 38.5–80.5 |
|  | **Lymphocytes** | % | 42.2 | 34.2 | 16.5–49.5 |
|  | **Monocytes** | % | 9.1 | 8 | 2–10 |
|  | **Eosinophils** | % | 1.2 | 1.1 | 0–8.5 |
|  | **Basophils** | % | 1 | 0.4 | 0–2.5 |
|  | **Hemoglobin** | g/dL | 13.2 | 13 | 13.7–16.8 |
|  | **Platelets** | x10⁴/µL | 225 | 265 | 158–348 |
| Biochemistry | **CRP** | mg/dL | 0.01 | 0.44 | < 0.14 |
|  | **Creatinine** | mg/dL | 0.99 | 1 | 0.65–1.07 |
| Immunology | **IgE** | IU/mL | N/A | 94 | 27.54-138.34 |
| Tumor Markers | **PSA** | ng/mL | 1.2 | 1.1 | < 4.0 |
